# Comparison of four multilocus sequence typing schemes and amino acid biosynthesis based on genomic analysis of *Bacillus subtilis*

**DOI:** 10.1371/journal.pone.0282092

**Published:** 2023-02-21

**Authors:** Gawon Lee, Sojeong Heo, Tao Kim, Hong-Eun Na, Jong-Hoon Lee, Do-Won Jeong

**Affiliations:** 1 Department of Food and Nutrition, Dongduk Women’s University, Seoul, Republic of Korea; 2 Department of Food Science and Biotechnology, Kyonggi University, Suwon, Republic of Korea; National Sun Yat-sen University, TAIWAN

## Abstract

*Bacillus subtilis*, a valuable industrial microorganism used in starter cultures in soybean fermentation, is a species of bacteria with interspecies diversity. Here, four multilocus sequence typing (MLST) schemes developed to assess the diversity of *B*. *subtilis* or *Bacillus* spp. were applied and compared to confirm the interspecies diversity of *B*. *subtilis*. In addition, we analyzed correlations between amino acid biosynthesis genes and sequence types (STs); this is important because amino acids are key taste components in fermented foods. On applying the four MLST methods to 38 strains and the type strain of *B*. *subtilis*, 30 to 32 STs were identified. The discriminatory power was 0.362–0.964 for the genes used in the MLST methods; the larger the gene, the greater the number of alleles and polymorphic sites. All four MLST methods showed a correlation between STs and strains that do not possess the *hutHUIG* operon (which contains genes required for the production of glutamate from histidine). This correlation was verified using 168 further genome-sequence strains.

## Introduction

*Bacillus subtilis* is isolated from several fermented soybean products of East Asia, including *meju* and *doenjang* from Korea, *douchi* from China, and *natto* and *miso* from Japan [1–5]. The long history of consumption of fermented foods is considered to be the history of consumption of the microorganisms involved in the food fermentation, so *B*. *subtilis* is a “generally recognized as safe” microorganism. During fermentation, *B*. *subtilis* produces amino acids, fatty acids, and volatile compounds from macromolecules such as starch and proteins via the activities of extracellular amylases and proteases [6–10]. In particular, peptides, amino acids, and their derivatives contribute to the taste of fermented foods [11] and such compounds produced by *B*. *subtilis* contribute to the quality and sensory properties of fermented soybean [8, 12]. For this reason, protease activities for amino acid production have been used as criteria for selection of fermentation starter strains [13, 14].

Amino acids play an important role in the sensory properties of foods, providing sweet, bitter, and umami tastes. Each amino acid has its own taste, and their combination contributes to the overall sensory properties of foods [15]. In particular, glutamic acid confers umami (savory) taste [16]. Although glutamic acid in proteins does not contribute umami taste, free amino acid produced from protein by proteolytic activity during fermentation does [17]. Microbial cultures that are involved in proteolytic processes during fermentation also contribute to organoleptic properties of the product foods, such as texture and flavor, and provide essential micronutrients such as minerals and vitamins [18, 19]. Using glutamic acid-producing strains as starter candidates might contribute to the enhancement of umami tastes and product food quality.

Multilocus sequence typing (MLST) is a highly discriminatory typing method appropriate for genetically coherent organisms that can provide a measure of genetic relatedness among strains [20]. MLST data have often been used to distinguish between strains of species that exhibit close phylogenetic relatedness. MLST based on concatenated DNA sequences of internal fragments of multiple housekeeping genes shows greater discriminatory power and reproducibility than phenotypic typing [21]. Five MLST methods have been developed for *Bacillus* spp., including *B*. *subtilis*, to help understand the correlations between sequence type (ST) and antibacterial, antifungal, bio-degradation, antibiotic resistance, and biofilm forming activities, respectively [22–26]. To define STs in an MLST scheme, multiple housekeeping genes (usually 7–9) are partially and accurately sequenced. For each gene used, the distinct sequences found within a species are considered different alleles. Each allele (i.e., each observed unique gene sequence) is assigned an “allele number.” The combination of allele numbers at the multiple loci used in the particular typing system defines the ST of the given strain. *Bacillus* spp. show much variation within their housekeeping genes, which means that individual strains can be typed by this method. However, there are no comparison results between the different MLST methods for *Bacillus*. Nor is there any data on correlations between STs determined by MLST and amino acid biosynthesis by *Bacillus* strains. The aims of this study were to compare the discriminatory ability of the previously developed MLST methods, and to investigate correlations between genetic differences and amino acid biosynthesis ability of *B*. *subtilis* strains.

## Materials and methods

### Bacterial strains and culture conditions

Thirty-eight *B*. *subtilis* isolates with previously published genome sequences in the GenBank database were kindly provided by the Microbial Institute for Fermentation Industry (S1 Table). All these strains were isolated from food in Korea. The taxonomic identities of all isolates were confirmed by sequence analysis of near-complete 16S rRNA gene regions [27], and all showed >99.9% identity to the type strain, *B*. *subtilis* NCIB 3610^T^. All *B*. *subtilis* isolates were cultured in tryptic soy agar (Difco, USA) and/or tryptic soy broth (Difco) at 37°C for 24 h.

### MLST schemes and data analysis

MLST scheme P1 for *B*. *subtilis* uses seven housekeeping genes: *glpF*, *ilvD*, *pta*, *purH*, *pycA*, *rpoD*, and *tpiA* [22] (Table 1). All isolates were assigned allelic profiles and STs according to the previously developed P1 scheme [22] (Table 1); partial gene sequences of housekeeping genes were retrieved from the genome sequences and the sequence at each location was compared with the known alleles from previous MLST results to specify the allele number. Then, the ST was derived from the combination of the allele numbers for all the genes used in the typing scheme. In schemes S2 and S3, nine and seven housekeeping genes are used, respectively [24, 25]. Scheme P1 provides information on allele numbers, nucleotide sequences, and ST profiles on the internet, but schemes S2 and S3 are not open-source, so we could not obtain exact information about alleles and STs for them. Thus, we newly designated allele numbers and STs for these schemes. In the case of scheme L1, a method previously developed for *B*. *licheniformis* and *B*. *paralicheniformis* [26], the same sequence as the allele identified at this time could not find the same nucleotide sequence as the alleles identified in the previous experiment, so ST was derived by newly assigning alleles following the previous allele number.

**Table 1:** Five MLST methods for *Bacillus* species.

Method	Concatenated order of genes for MLST	Target species	Purpose	Reference
P1	*glpF*, *ilvD*, *pta*, *purH*, *pycA*, *rpoD*, *tpiA*	*B*. *subtilis*	-	[22]
S1	*rpoD*, *glpF*, *ilvD*, *ptA*, *tpiA*, *pycA*, *purH*	*B*. *subtilis*	Probiotics and diversity	[23]
S2	*gyrA*, *gyrB*, *purH*, *glpF*, *pycA*, *ilvD*, *rpoD*, *tpiA*, *pta*	*B*. *subtilis*	Biocontrol features and diversity	[24]
S3	*gyrB*, *adk*, *pycA*, *pyrE*, *sucC*, *mutL*, *aroE*	*B*. *subtilis*	Antifungal properties and diversity	[25]
L1	*adk*, *ccpA*, *glpF*, *gmk*, *ilvD*, *pur*, *spo0A*, *tpi*	*B*. *paralicheniformis*, *B*. *licheniformis*	Classification between two species	[26]

Phylogenetic analysis based on STs was performed using the maximum likelihood method in MEGA 7.0 software. STs were analyzed to identify clonal complexes (CCs), which are related clusters of STs, using the eBURST algorithm to identify single-locus variants, double-locus variants, and singletons [28]. Singletons were defined as STs with at least two allelic mismatches with all other STs [28]. Typing efficiency is defined as the number of alleles per polymorphic site for each housekeeping gene [29]. Discriminatory power is the likelihood that two strains differentiate when randomly selected from a population of unrelated strains [29]. The non-synonymous (dN) and synonymous (dS) nucleotide substitutions per site were estimated using MEGA 7.0 software [30]. Using phyloviz software, minimum spanning trees were drawn from the alleles and STs determined by each scheme. The minimum spanning trees were constructed using the goeBURST Full MST algorithm, and the level is assigned the number of loci in the dataset [31].

### Comparison of genomes to assess amino acid biosynthesis

Genome sequence data for 39 *B*. *subtilis* strains was obtained from the NCBI database (http://ncbi.nlm.nih.gov/genomes; S1 Table). For metabolic pathway analysis based on protein coding genes, genome sequences of 39 strains were uploaded to the Rapid Annotations using Subsystems Technology (RAST) server for SEED-based automated annotation, and subjected to whole-genome sequence-based comparative analysis and Kyoto Encyclopedia of Genes and Genomes pathway analysis [32]. The generated metabolic pathways of the strains were verified using the iPath module (ver. 3) [33]. The Efficient Database framework for comparative Genome Analyses using BLAST score Ratios (EDGAR) was used for core genome, pan-genome, and singleton analyses [34]. Further comparative analyses were performed for specific regions and genes of interest using the BLASTN, BLASTX, and BLASTP tools.

## Results and discussion

### Comparison of four MLST schemes

Four MLST schemes have been developed to evaluate the genetic diversity of *B*. *subtilis*: schemes P1 and S1–S3 (Table 1) [22–25]. The MLST schemes are built on partial sequences of 7–9 housekeeping genes (Table 1). Among them, the types and fragments of the housekeeping genes used are the same in schemes S1 and P1; only the order of concatenation is different. Thus, scheme S1 was not used in this study. Also, we previously developed an MLST method for *B*. *licheniformis* (scheme L1), which was successfully applied to genotype analysis of *B*. *subtilis* [26, 35]. This scheme was also applied in the present work.

**Fig 1:**
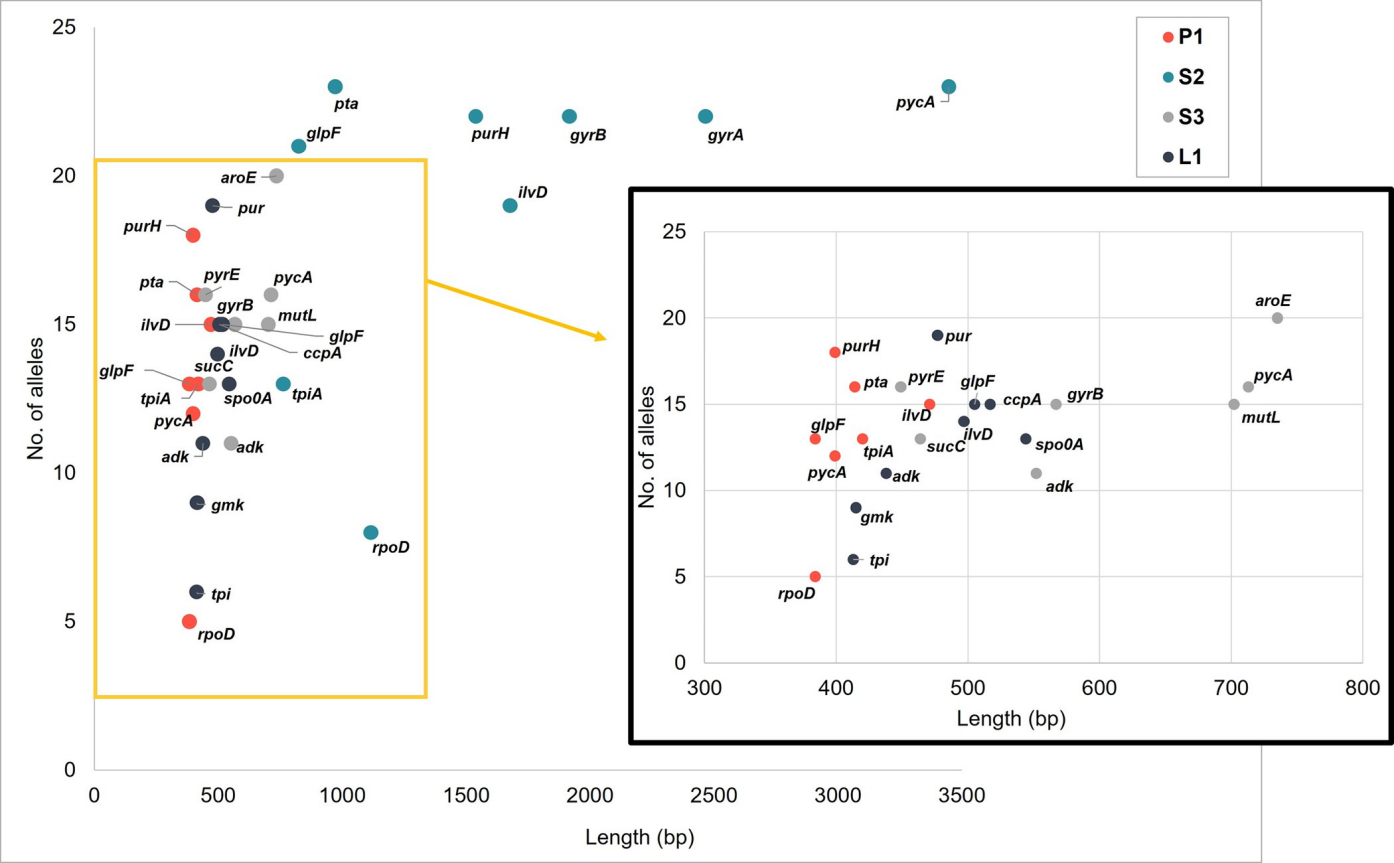
Scatterplot comparing genes used in multilocus sequence typing (MLST) methods for *Bacillus subtilis*. Comparison of housekeeping gene lengths and variability in terms of number of alleles for 39 strains of *B*. *subtilis*.

Genome sequences of 39 *B*. *subtilis* strains were retrieved from the GenBank database and then analyzed for alleles and ST according to each of the MLST schemes (S2 Table). Except for scheme P1, updated information on allele and ST was not known, so a new number was assigned. Scheme S2 gave the greatest diversity of genetic classification. The size of the housekeeping genes used in scheme S2 is more than double that used in the other schemes (Fig 1). After dividing the number of alleles by DNA size, simple calculating the strain per base, scheme P1 seemed to indicate the greatest genetic diversity.

**Fig 2:**
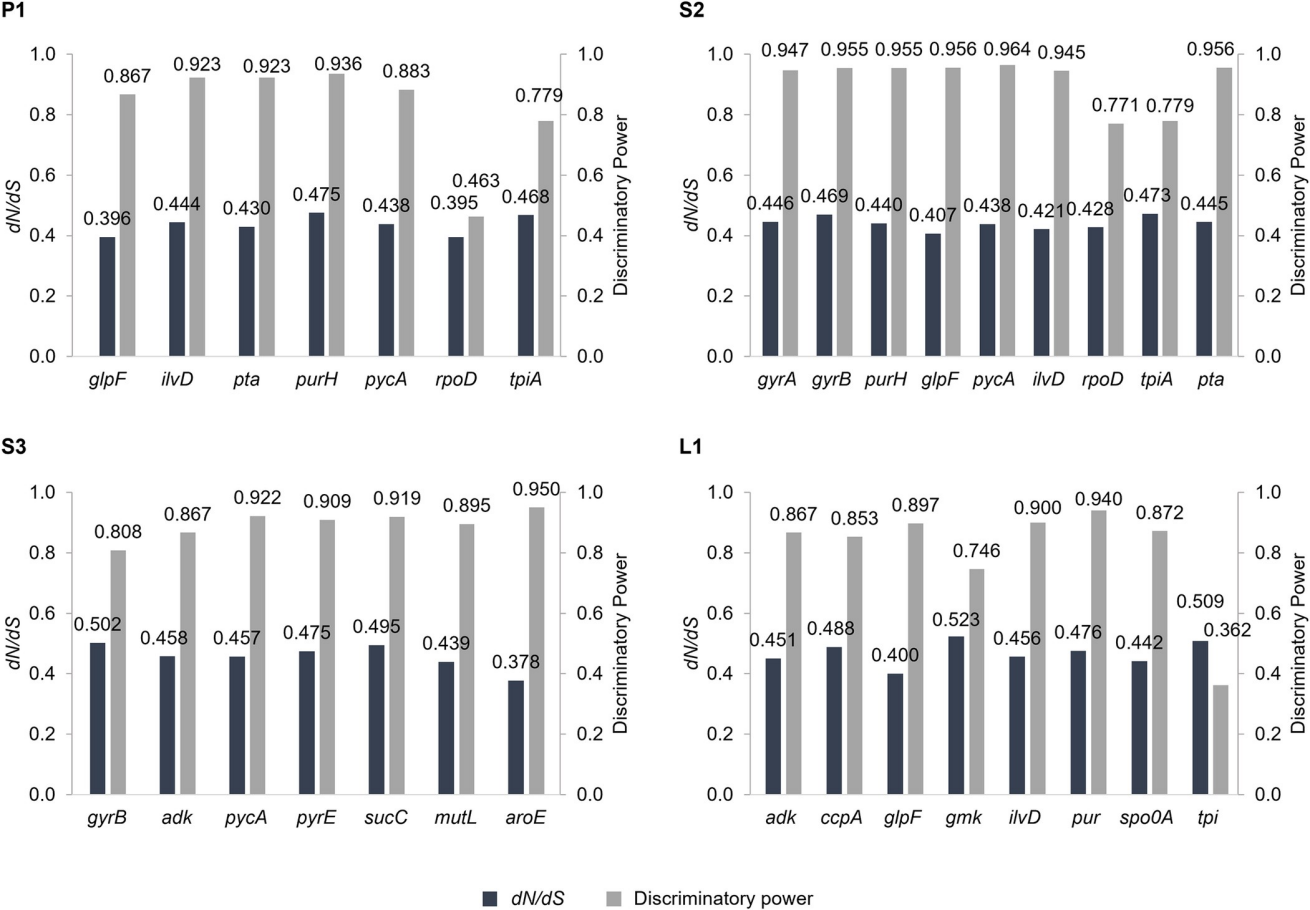
Non-synonymous nucleotide substitutions per site/synonymous nucleotide substitutions per site (*dN/dS*) values and discriminatory power of four MLST schemes.

The allelic variation was analyzed for each gene sequence. The number of polymorphic sites within each gene ranged from 4 (in *rpoD*, which is used in scheme P1) to 117 (in *purH*, used in scheme S2), and the number of alleles ranged from 5 (for *rpoD*) to 23 (for *pycA* and *pta*, used in scheme S2) (S3 Table). Although the number of polymorphic sites varied, the dN/dS ratio for each housekeeping gene showed no significant difference. The average dN/dS ratio and discriminatory power across all MLST genes was 0.4503 and 0.86, respectively (Fig 2 and S3 Table). The discriminatory power was 0.362–0.964 for the genes used in the MLST methods; the larger the gene, the greater the number of alleles and polymorphic sites. When comparing MLST schemes, scheme S3 showed that the highest nucleotide diversity, and this scheme generated 30 STs. However, two schemes (P1 and L1) and scheme S2 generated 31 and 32 STs from the 39 strains, respectively. Based on the above results, scheme S2 gave the greatest diversity of alleles and polymorphic sites. However, we determined that scheme P1 was better because the difference in diversity between S2 and P1 was not significant, and scheme P1 is already publicly accessible. If other housekeeping genes with higher discriminatory power were used for a modified scheme P1 in place of *rpoD* and/or *tpiA* (which have low discriminatory power), the scheme might identify greater genetic diversity.

### Genomic analysis of amino acid biosynthesis in *B*. *subtilis*

**Fig 3:**
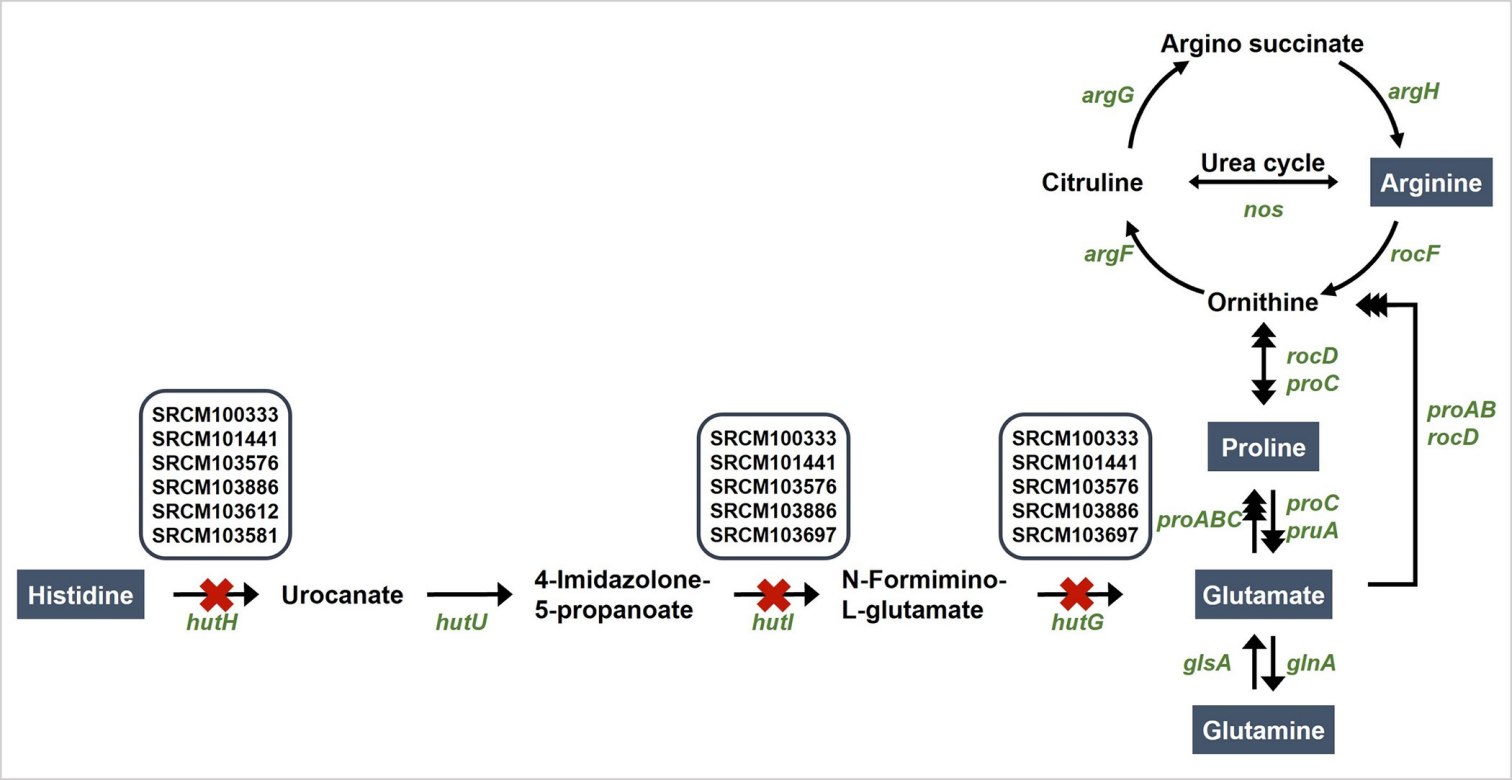
Biosynthesis pathway from histidine to glutamate in *B*. *subtilis*. Red crosses indicate strains that do not possess *hutH*, *hutI*, and/or *hutG*. Genes and amino acids are depicted in green italics and blue boxes, respectively. The black arrows correspond to potential enzymatic reactions catalyzed by gene products encoded in the genome of *B*. *subtilis*.

To identify correlations between STs and amino acid production, first, *in silico* prediction of the amino acid biosynthesis pathways in *B*. *subtilis* was performed (Fig 3) using the complete genome sequences of the 38 *B*. *subtilis* strains and the type strain that were analyzed above. Thirty-one of the *B*. *subtilis* strains and the type strain appeared to contain loci coding for all the enzymes required for the biosynthesis of all amino acids. However, seven of the strains did not encode two or more of the biosynthesis genes *hutH*, *hutI*, and *hutG* that are required for conversion of histidine to glutamate. Glutamate is a precursor of glutamine, arginine, and proline; thus, these strains could not synthesize these amino acids *de novo*. However, the genes for biosynthesis of arginine, glutamine, and arginine from glutamate were retained, so those amino acids could be synthesized if glutamate were provided from outside the cell (Fig 3).

### Amino acid biosynthesis profiles of *B*. *subtilis* isolates grouped by ST

To examine correlations between the amino acid production-gene patterns and STs, we compared the STs and amino acid biosynthesis components using phylogenetic trees (S1 Fig). All the schemes were able to classify strains that did not possess genes for production of glutamate from histidine into STs and there was no significant difference between them, so scheme P1 was applied going forward. Using scheme P1, the seven strains lacking glutamate biosynthesis genes were identified into six STs: ST208 (strains SRCM101441 and SRCM103886), ST210 (SRCM103576), ST211 (SRCM103581), ST213 (SRCM100333), ST236 (SRCM103612), and ST240 (SRCM103697).

**Fig 4:**
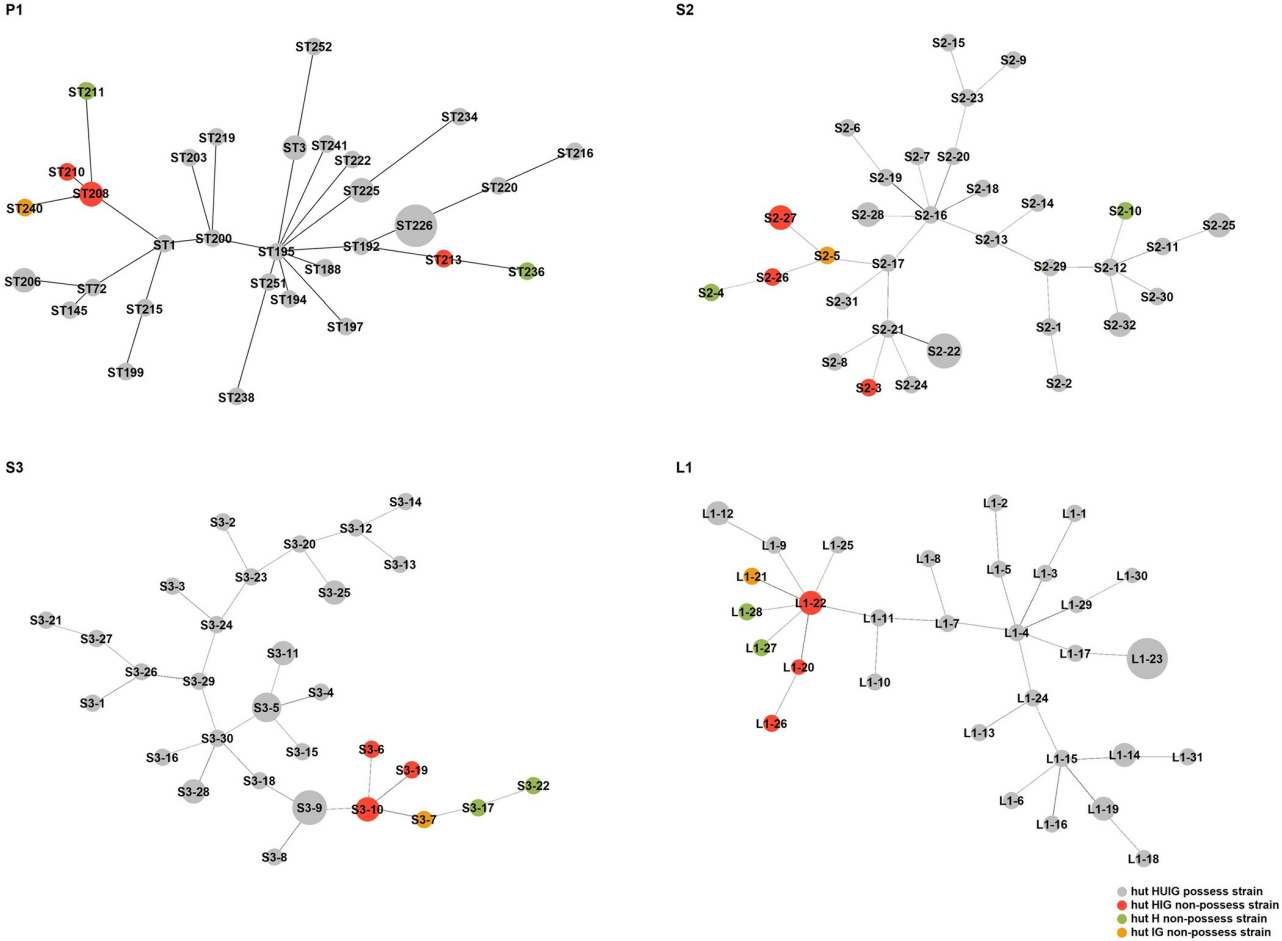
Minimum spanning trees for sequence types generated by four MLST methods. Numbers in the trees indicate STs. Circle sizes are proportional to the number of isolates belonging to that ST.

Fig 4 shows minimum spanning trees illustrating predicted genetic relationships among the 39 strains based on the four MLST schemes. Strains that do not possess the *hutHUIG* operon were distinguished from those that do, and the strains lacking these genes clustered together in the trees. It can be seen that S3 and L1 are bound from S3-10 and L1-22.

To confirm the correlation between apparent ability to synthesize glutamate from histidine and ST, we analyzed the presence of four genes, *hutH*, *hutU*, *hutI*, and *hutG*, involved in glutamic acid synthesis in *B*. *subtilis* genomes in public databases as of July 2022 (Table 2). By that time, 207 *B*. *subtilis* genomes had been registered (the 39 we analyzed above, plus another 168). Based on analysis of the genomes, 26 of the 168 strains did not possess *hutG* and *hutI*, and 28 of the strains did not possess *hutH*. Six strains among the 168 *B*. *subtilis* strains corresponded to ST208, and none of them had *hutH*, *hutI*, and *hutG* genes. Seventeen strains corresponded to ST4, which also lacked these three genes. The remaining six strains corresponded to different STs, among which new STs were included (strains DKU_NT_02, BS38, MH-1, PJ-7, JAAA, and FX-1 were assigned to ST207, ST209, ST212, ST214, ST245, and ST256, respectively). The data for ST208 and ST4 showed that there is a correlation between the absence of the genes for glutamate synthesis and ST. In the case of other STs, it will only be possible to draw conclusions when further data (i.e., more sequences) are accumulated.

**Table 2:** Presence of genes involved in potential glutamate production from histidine (analysis of data available as of July 2022).

	No. of strains	No of *hutHUIG*-possessing strains			
		*hutH*	*hutU*	*hutI*	*hutG*
SRCM strains and type strain	39	33	39	34	34
Other strains	168	140	168	142	142
Total^a^	207	173	207	176	176

SRCM strains mean strains provided by the Microbial Institute for Fermentation Industry.

^a^ Number of registered genomes as of July 2022.

## General discussion

MLST is a useful tool for identifying genetic diversity within species [21]. Studies have investigated correlations between MLST and the presence or absence of virulence factors, differences between regions, and the presence or absence of antibiotic resistance [36–40]. Five MLST methods have been developed so far for *B*. *subtilis* and genus *Bacillus*. In this study, comparative analysis was performed between four of these MLST methods (omitting scheme S1) [22–26]. In addition, relationships between the amino acid biosynthesis genes of *B*. *subtilis* and the STs derived from MLST were analyzed.

MLST scheme P1 has the advantage of being available anywhere in the world because it uses a public database [22]. It is method of identifying genetic diversity based on seven housekeeping genes. In this study, MLST using scheme P1 derived 31 STs from 39 *B*. *subtilis* strains; this result was similar to that obtained using the other three MLST methods (S1 Fig). It was shown that six STs did not possess the *hutHUIG* operon, which encodes enzymes that produce glutamate from histidine; thus, there appears to be a correlation between amino acid biosynthesis and ST in *B*. *subtilis*. The *rpoD* gene used in scheme P1 is highly conserved (having few polymorphic sites), resulting in a low discriminatory power. If that gene were to be replaced with a gene that exhibits higher discriminatory power, more effective diversity results could be achieved using the modified scheme P1.

Scheme S2 was developed to compare the genotype with the secondary metabolites of *B*. *subtilis* [24]. MLST by scheme S2 uses nine housekeeping genes, and the entire housekeeping gene sequences are used; thus, the average gene size is about 1.6 kb, compared with about 500 bp in the other schemes. As a result, the genetic diversity appears greater than that indicated by the other MLST schemes. Nevertheless, the number of STs produced by scheme S2 was 32 (compared with 30 or 31 by the other methods), which indicated that the number and size of the genes used for typing did not contribute much to the genotyping. The discriminatory power of scheme S2 was higher than that of the other methods.

Scheme S3, which includes seven genes for typing, was developed to identify the *sfp* gene, which is responsible for the production of a lipopeptide biosurfactant, and genetic diversity in *Bacillus* species [25]. Using this scheme, 30 STs were derived from the 39 strains. Although this scheme showed the correlation between *hutHUIG* operon-free strains and ST, it did not show superiority over other methods.

MLST scheme L1 is a method built to distinguish between *B*. *licheniformis* and *B*. *paralicheniformis*, which have high genetic homogeneity [26]. It was expanded to re-identify *B*. *velezensis* [41], or *B*. *subtilis* from other *Bacillus* species [35]. Here, it was applied to identify genetic diversity within *B*. *subtilis*, and results similar to those from the other MLST methods were obtained. This scheme discriminated strains according to the presence or absence of the *hutHUIG* operon. However, the scheme uses genes *gmk* and *tpi* for typing—these genes show low polymorphism, so we suggest that more effective results could be obtained if genes with greater discriminatory power were used instead.

In conclusion, comparing four MLST schemes for *B*. *subtilis* shows that no one method is obviously superior to the others. However, scheme P1 has a significant advantage because it is already a well-established and publicly accessible system. We determined that there are correlations between ST and amino acid biosynthesis genes in *B*. *subtilis*. If further research is conducted, MLST can be used to secure desired amino acid-producing strains.

## Supporting information

S1 Fig Phylogenetic trees for the four MLST schemes. The data were compared using simple matching coefficients and were clustered by the maximum likelihood method. Branches with bootstrap values of <50% have been collapsed. The scale the diagram is the pairwise distance expressed as the percentage dissimilarity.
(DOCX)

S1 Table Bacterial strains and genome accession numbers. (DOCX)

S2 Table Sequence types of *Bacillus subtilis* using four MLST schemes, as well as the numbers of alleles. (DOCX)

S3 Table Compositional characteristics of genes used in the four MLST schemes for *B*. *subtilis*. (DOCX)
