# Supplementary material for: Comparison of four multilocus sequence typing schemes and amino acid biosynthesis based on genomic analysis of Bacillus subtilis
Source: PLoS One. 2023 Feb 21;18(2):e0282092. doi: 10.1371/journal.pone.0282092 (PMC9943010; doi:10.1371/journal.pone.0282092)

**S1 Fig. Phylogenetic tree of developed four MLST scheme.** The data were compared using simple matching coefficients and were clustered by the maximum likelihood method. Branches with bootstrap values of 50% have been collapsed. The scale the diagram is the pairwise distance expressed as the percentage of dissimilarity.


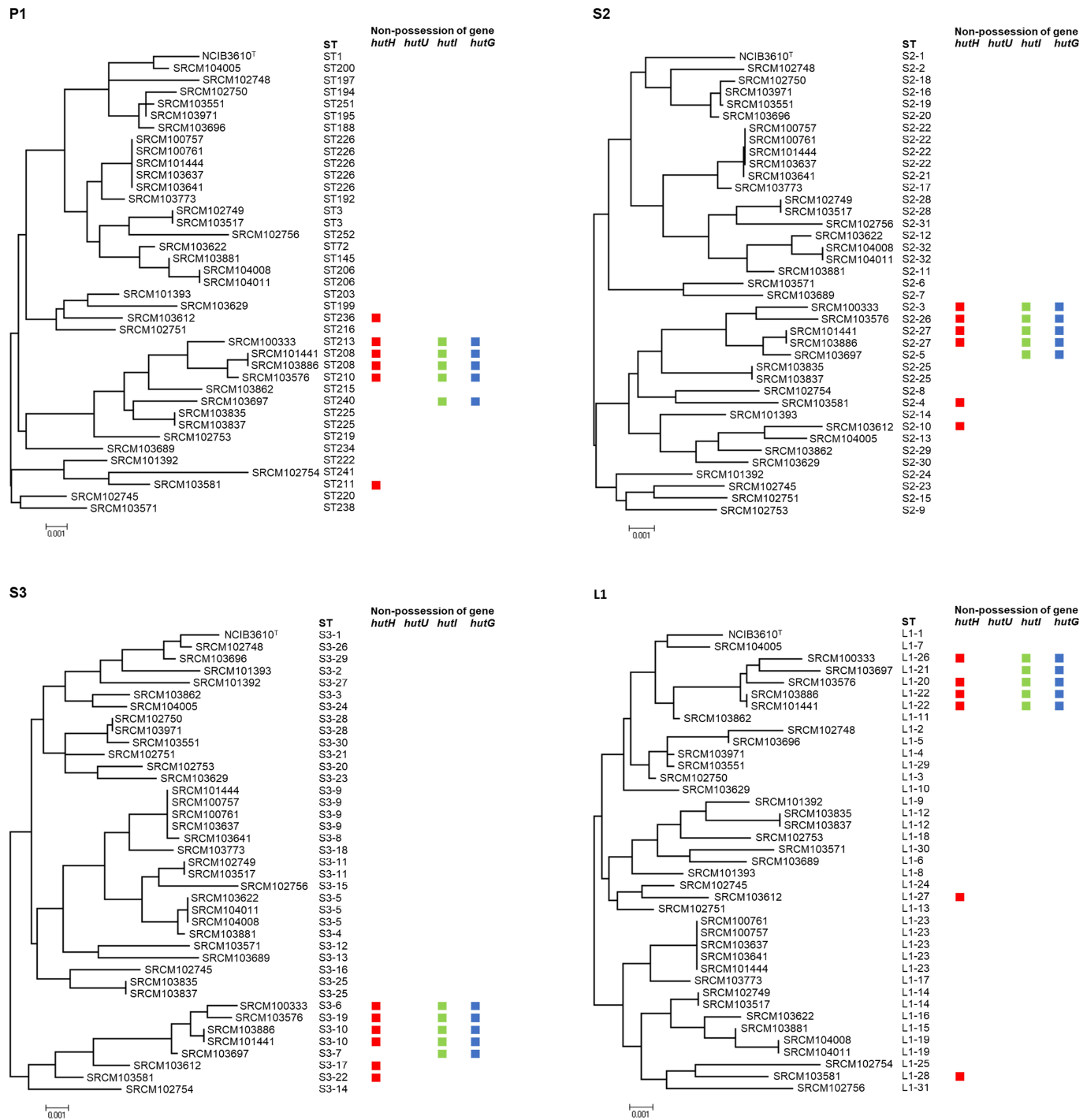

Supplement: S1 Fig — The data were compared using simple matching coefficients and were clustered by the maximum likelihood method. Branches with bootstrap values of <50% have been collapsed. The scale the diagram is the pairwise distance expressed as the percentage dissimilarity. (DOCX) [file pone.0282092.s001.docx]
