# Supplementary material for: Comparison of four multilocus sequence typing schemes and amino acid biosynthesis based on genomic analysis of Bacillus subtilis
Source: PLoS One. 2023 Feb 21;18(2):e0282092. doi: 10.1371/journal.pone.0282092 (PMC9943010; doi:10.1371/journal.pone.0282092)
Supplement: S1 Table — (DOCX) [file pone.0282092.s002.docx]

**S1 Table. Bacterial strains and genome accession numbers.**

| **No.** | **species** | **strain** | **origin** | **Accession Number** |
| --- | --- | --- | --- | --- |
| 1 | *Bacillus subtilis* | NCIB3610^T^ | Raw milk | NZ_CP020102 |
| 2 | *Bacillus subtilis* | SRCM100333 | food | NZ_CP021892 |
| 3 | *Bacillus subtilis* | SRCM100757 | food | NZ_CP021499 |
| 4 | *Bacillus subtilis* | SRCM100761 | food | NZ_CP021889 |
| 5 | *Bacillus subtilis* | SRCM101392 | food | NZ_CP021921 |
| 6 | *Bacillus subtilis* | SRCM101393 | gochujang | NZ_CP031693 |
| 7 | *Bacillus subtilis* | SRCM101441 | food | NZ_CP021507 |
| 8 | *Bacillus subtilis* | SRCM101444 | food | NZ_CP021498 |
| 9 | *Bacillus subtilis* | SRCM102745 | kimchi | NZ_CP028209 |
| 10 | *Bacillus subtilis* | SRCM102748 | kimchi | NZ_CP028212 |
| 11 | *Bacillus subtilis* | SRCM102749 | kimchi | NZ_CP028213 |
| 12 | *Bacillus subtilis* | SRCM102750 | kimchi | NZ_CP028215 |
| 13 | *Bacillus subtilis* | SRCM102751 | kimchi | NZ_CP028217 |
| 14 | *Bacillus subtilis* | SRCM102753 | doenjang | NZ_CP028201 |
| 15 | *Bacillus subtilis* | SRCM102754 | doenjang | NZ_CP028202 |
| 16 | *Bacillus subtilis* | SRCM102756 | ganjang | NZ_CP028218 |
| 17 | *Bacillus subtilis* | SRCM103517 | food | NZ_CP035226 |
| 18 | *Bacillus subtilis* | SRCM103551 | food | NZ_CP035230 |
| 19 | *Bacillus subtilis* | SRCM103571 | food | NZ_CP035231 |
| 20 | *Bacillus subtilis* | SRCM103576 | food | NZ_CP035402 |
| 21 | *Bacillus subtilis* | SRCM103581 | food | NZ_CP035403 |
| 22 | *Bacillus subtilis* | SRCM103612 | food | NZ_CP035406 |
| 23 | *Bacillus subtilis* | SRCM103622 | food | NZ_CP035411 |
| 24 | *Bacillus subtilis* | SRCM103629 | food | NZ_CP035413 |
| 25 | *Bacillus subtilis* | SRCM103637 | food | NZ_CP035414 |
| 26 | *Bacillus subtilis* | SRCM103641 | food | NZ_CP035390 |
| 27 | *Bacillus subtilis* | SRCM103689 | food | NZ_CP035391 |
| 28 | *Bacillus subtilis* | SRCM103696 | food | NZ_CP035394 |
| 29 | *Bacillus subtilis* | SRCM103697 | food | NZ_CP035395 |
| 30 | *Bacillus subtilis* | SRCM103773 | food | NZ_CP035397 |
| 31 | *Bacillus subtilis* | SRCM103835 | food | NZ_CP035400 |
| 32 | *Bacillus subtilis* | SRCM103837 | food | NZ_CP035401 |
| 33 | *Bacillus subtilis* | SRCM103862 | food | NZ_CP035161 |
| 34 | *Bacillus subtilis* | SRCM103881 | food | NZ_CP035165 |
| 35 | *Bacillus subtilis* | SRCM103886 | food | NZ_CP035162 |
| 36 | *Bacillus subtilis* | SRCM103971 | food | NZ_CP035166 |
| 37 | *Bacillus subtilis* | SRCM104005 | food | NZ_CP035164 |
| 38 | *Bacillus subtilis* | SRCM104008 | food | NZ_CP035167 |
| 39 | *Bacillus subtilis* | SRCM104011 | food | NZ_CP035191 |
