# Supplementary material for: Comparison of four multilocus sequence typing schemes and amino acid biosynthesis based on genomic analysis of Bacillus subtilis
Source: PLoS One. 2023 Feb 21;18(2):e0282092. doi: 10.1371/journal.pone.0282092 (PMC9943010; doi:10.1371/journal.pone.0282092)
Supplement: S2 Table — (DOCX) [file pone.0282092.s003.docx]

**S2 Table. Sequence types of the *Bacillus subtilis* using four MLST schemes, as well as the numbers of alleles.**

| P1 | | | | | | | | | | |
| --- | --- | --- | --- | --- | --- | --- | --- | --- | --- | --- |
| Sequence  Type (ST) | Strain | Allelic profile | | | | | | | | |
|  |  | *glpF* | *ilvD* | *pta* | *purH* | *pycA* | *rpoD* | *tpiA* |  |  |
| 1 | NCIB3610^T^ | 1 | 1 | 1 | 1 | 1 | 1 | 1 |  |  |
| 3 | SRCM102749, SRCM103517 | 3 | 3 | 2 | 3 | 2 | 1 | 1 |  |  |
| 72 | SRCM103622 | 1 | 48 | 55 | 56 | 34 | 1 | 1 |  |  |
| 145 | SRCM103881 | 59 | 48 | 55 | 56 | 34 | 1 | 1 |  |  |
| 188 | SRCM103696 | 2 | 104 | 20 | 4 | 1 | 1 | 1 |  |  |
| 192 | SRCM103773 | 3 | 2 | 4 | 4 | 34 | 1 | 3 |  |  |
| 194 | SRCM102750 | 1 | 89 | 4 | 4 | 1 | 1 | 2 |  |  |
| 195 | SRCM103971 | 2 | 89 | 4 | 4 | 1 | 1 | 1 |  |  |
| 197 | SRCM102748 | 52 | 57 | 1 | 63 | 1 | 1 | 33 |  |  |
| 199 | SRCM103629 | 1 | 57 | 44 | 38 | 26 | 1 | 1 |  |  |
| 200 | SRCM104005 | 2 | 1 | 5 | 1 | 1 | 1 | 1 |  |  |
| 203 | SRCM101393 | 4 | 1 | 5 | 38 | 3 | 1 | 2 |  |  |
| 206 | SRCM104008, SRCM104011 | 36 | 48 | 5 | 56 | 34 | 41 | 1 |  |  |
| 208 | SRCM101441, SRCM103886 | 1 | 1 | 2 | 6 | 4 | 3 | 1 |  |  |
| 210 | SRCM103576 | 1 | 2 | 2 | 6 | 4 | 3 | 1 |  |  |
| 211 | SRCM103581 | 1 | 65 | 35 | 86 | 56 | 3 | 49 |  |  |
| 213 | SRCM100333 | 3 | 111 | 4 | 6 | 4 | 1 | 4 |  |  |
| 215 | SRCM103862 | 1 | 1 | 108 | 119 | 26 | 1 | 1 |  |  |
| 216 | SRCM102751 | 61 | 105 | 5 | 120 | 56 | 1 | 82 |  |  |
| 219 | SRCM102753 | 101 | 64 | 5 | 121 | 84 | 1 | 56 |  |  |
| 220 | SRCM102745 | 61 | 105 | 20 | 122 | 100 | 1 | 56 |  |  |
| 222 | SRCM101392 | 99 | 65 | 109 | 123 | 26 | 1 | 64 |  |  |
| 225 | SRCM103835, SRCM103837 | 59 | 18 | 110 | 125 | 101 | 1 | 1 |  |  |
| 226 | SRCM103641, SRCM100757,  SRCM100761, SRCM101444,  SRCM103637 | 3 | 2 | 20 | 122 | 34 | 1 | 3 |  |  |
| 234 | SRCM103689 | 98 | 3 | 61 | 126 | 101 | 82 | 84 |  |  |
| 236 | SRCM103612 | 3 | 107 | 112 | 4 | 4 | 3 | 4 |  |  |
| 238 | SRCM103571 | 32 | 65 | 43 | 133 | 55 | 41 | 87 |  |  |
| 240 | SRCM103697 | 36 | 1 | 112 | 6 | 4 | 1 | 1 |  |  |
| 241 | SRCM102754 | 54 | 112 | 113 | 130 | 107 | 1 | 88 |  |  |
| 251 | SRCM103551 | 2 | 89 | 4 | 4 | 1 | 1 | 87 |  |  |
| 252 | SRCM102756 | 3 | 114 | 36 | 135 | 2 | 60 | 91 |  |  |
| S2 | | | | | | | | | | |
| Sequence  Type (ST) | Strain | Allelic profile | | | | | | | | |
|  |  | *gyrA* | *gyrB* | *purH* | *glpF* | *pycA* | *ilvD* | *rpoD* | *tpiA* | *pta* |
| S2-1 | NCIB3610^T^ | 1 | 1 | 1 | 1 | 1 | 1 | 1 | 1 | 1 |
| S2-2 | SRCM102748 | 10 | 6 | 8 | 8 | 23 | 4 | 1 | 7 | 1 |
| S2-3 | SRCM100333 | 5 | 18 | 14 | 16 | 16 | 16 | 5 | 12 | 2 |
| S2-4 | SRCM103581 | 7 | 19 | 16 | 21 | 15 | 2 | 3 | 2 | 3 |
| S2-5 | SRCM103697 | 15 | 11 | 12 | 2 | 14 | 19 | 5 | 1 | 4 |
| S2-6 | SRCM103571 | 21 | 15 | 10 | 5 | 11 | 6 | 4 | 13 | 5 |
| S2-7 | SRCM103689 | 19 | 12 | 9 | 12 | 2 | 5 | 6 | 6 | 6 |
| S2-8 | SRCM102754 | 20 | 16 | 13 | 13 | 9 | 15 | 5 | 8 | 7 |
| S2-9 | SRCM102753 | 14 | 17 | 6 | 6 | 20 | 13 | 1 | 9 | 8 |
| S2-10 | SRCM103612 | 3 | 10 | 2 | 4 | 17 | 17 | 3 | 12 | 9 |
| S2-11 | SRCM103881 | 18 | 2 | 4 | 9 | 8 | 12 | 1 | 1 | 10 |
| S2-12 | SRCM103622 | 3 | 10 | 4 | 19 | 8 | 12 | 1 | 1 | 10 |
| S2-13 | SRCM104005 | 3 | 10 | 20 | 10 | 22 | 1 | 1 | 1 | 11 |
| S2-14 | SRCM101393 | 9 | 7 | 21 | 7 | 12 | 1 | 5 | 11 | 11 |
| S2-15 | SRCM102751 | 8 | 21 | 11 | 15 | 3 | 14 | 1 | 3 | 12 |
| S2-16 | SRCM103971 | 11 | 9 | 17 | 11 | 22 | 8 | 1 | 1 | 13 |
| S2-17 | SRCM103773 | 15 | 11 | 17 | 17 | 6 | 9 | 5 | 4 | 13 |
| S2-18 | SRCM102750 | 11 | 9 | 17 | 1 | 22 | 8 | 1 | 11 | 13 |
| S2-19 | SRCM103551 | 11 | 9 | 17 | 11 | 22 | 8 | 1 | 13 | 13 |
| S2-20 | SRCM103696 | 11 | 9 | 17 | 11 | 22 | 18 | 1 | 1 | 14 |
| S2-21 | SRCM103641 | 15 | 8 | 19 | 18 | 6 | 9 | 5 | 4 | 14 |
| S2-22 | SRCM100757, SRCM100761,  SRCM101444, SRCM103637 | 15 | 8 | 19 | 18 | 7 | 9 | 5 | 4 | 14 |
| S2-23 | SRCM102745 | 4 | 20 | 18 | 15 | 10 | 14 | 1 | 9 | 14 |
| S2-24 | SRCM101392 | 13 | 13 | 3 | 14 | 18 | 7 | 5 | 10 | 15 |
| S2-25 | SRCM103835, SRCM103837 | 12 | 4 | 5 | 9 | 21 | 3 | 1 | 1 | 16 |
| S2-26 | SRCM103576 | 6 | 14 | 12 | 20 | 14 | 9 | 3 | 1 | 17 |
| S2-27 | SRCM101441, SRCM103886 | 16 | 11 | 12 | 20 | 13 | 19 | 2 | 1 | 18 |
| S2-28 | SRCM102749, SRCM103517 | 22 | 22 | 22 | 17 | 5 | 11 | 8 | 1 | 19 |
| S2-29 | SRCM103862 | 3 | 10 | 7 | 19 | 18 | 1 | 1 | 1 | 20 |
| S2-30 | SRCM103629 | 3 | 10 | 21 | 19 | 19 | 4 | 5 | 1 | 21 |
| S2-31 | SRCM102756 | 17 | 5 | 15 | 17 | 4 | 10 | 7 | 5 | 22 |
| S2-32 | SRCM104008, SRCM104011 | 2 | 3 | 4 | 3 | 8 | 12 | 4 | 1 | 23 |
| S3 | | | | | | | | | | |
| Sequence Type (ST) | Strain | Allelic profile | | | | | | | | |
|  |  | *gyrB* | *adk* | *pycA* | *pyrE* | *sucC* | *mutL* | *aroE* |  |  |
| S3-1 | NCIB3610^T^ | 1 | 1 | 1 | 1 | 1 | 1 | 1 |  |  |
| S3-2 | SRCM101393 | 9 | 10 | 4 | 11 | 1 | 3 | 1 |  |  |
| S3-3 | SRCM103862 | 9 | 7 | 2 | 10 | 8 | 6 | 1 |  |  |
| S3-4 | SRCM103881 | 10 | 5 | 9 | 2 | 7 | 12 | 2 |  |  |
| S3-5 | SRCM103622, SRCM104008, SRCM104011 | 9 | 5 | 9 | 2 | 7 | 12 | 3 |  |  |
| S3-6 | SRCM100333 | 7 | 3 | 12 | 7 | 2 | 15 | 4 |  |  |
| S3-7 | SRCM103697 | 9 | 3 | 11 | 14 | 12 | 15 | 4 |  |  |
| S3-8 | SRCM103641 | 8 | 9 | 8 | 4 | 3 | 14 | 5 |  |  |
| S3-9 | SRCM100757, SRCM100761,  SRCM101444, SRCM103637 | 8 | 9 | 8 | 7 | 3 | 15 | 5 |  |  |
| S3-10 | SRCM101441, SRCM103886 | 9 | 3 | 11 | 7 | 12 | 15 | 5 |  |  |
| S3-11 | SRCM102749, SRCM103517 | 15 | 5 | 9 | 3 | 11 | 13 | 6 |  |  |
| S3-12 | SRCM103571 | 5 | 1 | 7 | 2 | 9 | 3 | 7 |  |  |
| S3-13 | SRCM103689 | 14 | 11 | 7 | 13 | 9 | 5 | 8 |  |  |
| S3-14 | SRCM102754 | 5 | 8 | 3 | 8 | 13 | 10 | 9 |  |  |
| S3-15 | SRCM102756 | 4 | 4 | 8 | 3 | 7 | 12 | 10 |  |  |
| S3-16 | SRCM102745 | 3 | 5 | 6 | 6 | 1 | 2 | 11 |  |  |
| S3-17 | SRCM103612 | 9 | 7 | 15 | 14 | 10 | 9 | 12 |  |  |
| S3-18 | SRCM103773 | 9 | 9 | 8 | 15 | 3 | 15 | 13 |  |  |
| S3-19 | SRCM103576 | 2 | 3 | 11 | 7 | 12 | 15 | 13 |  |  |
| S3-20 | SRCM102753 | 12 | 1 | 10 | 12 | 11 | 3 | 14 |  |  |
| S3-21 | SRCM102751 | 11 | 6 | 13 | 16 | 6 | 8 | 15 |  |  |
| S3-22 | SRCM103581 | 6 | 2 | 14 | 14 | 10 | 11 | 15 |  |  |
| S3-23 | SRCM103629 | 9 | 7 | 4 | 12 | 8 | 3 | 16 |  |  |
| S3-24 | SRCM104005 | 9 | 7 | 16 | 5 | 8 | 6 | 17 |  |  |
| S3-25 | SRCM103835, SRCM103837 | 13 | 1 | 5 | 7 | 11 | 4 | 18 |  |  |
| S3-26 | SRCM102748 | 9 | 1 | 16 | 1 | 1 | 1 | 19 |  |  |
| S3-27 | SRCM101392 | 11 | 1 | 2 | 9 | 5 | 7 | 19 |  |  |
| S3-28 | SRCM102750, SRCM103971 | 9 | 5 | 16 | 15 | 4 | 1 | 20 |  |  |
| S3-29 | SRCM103696 | 9 | 1 | 16 | 1 | 1 | 6 | 20 |  |  |
| S3-30 | SRCM103551 | 9 | 5 | 16 | 15 | 3 | 15 | 20 |  |  |
| L1 | | | | | | | | | | |
| Sequence Type (ST) | Strain | Allelic profile | | | | | | | | |
|  |  | *adk* | *ccpA* | *glpF* | *gmk* | *ilvD* | *pur* | *spo0A* | *tpi* |  |
| L1-1 | NCIB3610^T^ | 4 | 6 | 6 | 7 | 9 | 8 | 8 | 4 |  |
| L1-2 | SRCM102748 | 4 | 11 | 11 | 7 | 18 | 14 | 8 | 4 |  |
| L1-3 | SRCM102750 | 7 | 10 | 6 | 10 | 9 | 17 | 8 | 4 |  |
| L1-4 | SRCM103971 | 7 | 9 | 14 | 10 | 9 | 17 | 8 | 4 |  |
| L1-5 | SRCM103696 | 4 | 10 | 14 | 7 | 18 | 17 | 8 | 4 |  |
| L1-6 | SRCM103689 | 14 | 18 | 15 | 15 | 14 | 23 | 8 | 4 |  |
| L1-7 | SRCM104005 | 9 | 9 | 14 | 12 | 9 | 15 | 9 | 4 |  |
| L1-8 | SRCM101393 | 13 | 12 | 10 | 11 | 9 | 26 | 9 | 4 |  |
| L1-9 | SRCM101392 | 4 | 9 | 17 | 13 | 19 | 16 | 10 | 4 |  |
| L1-10 | SRCM103629 | 9 | 9 | 20 | 11 | 18 | 26 | 10 | 4 |  |
| L1-11 | SRCM103862 | 9 | 9 | 20 | 13 | 9 | 20 | 11 | 4 |  |
| L1-12 | SRCM103835, SRCM103837 | 4 | 9 | 13 | 13 | 17 | 9 | 12 | 4 |  |
| L1-13 | SRCM102751 | 8 | 6 | 18 | 11 | 9 | 24 | 13 | 4 |  |
| L1-14 | SRCM102749, SRCM103517 | 7 | 19 | 19 | 11 | 15 | 8 | 14 | 4 |  |
| L1-15 | SRCM103881 | 7 | 19 | 13 | 11 | 14 | 25 | 14 | 4 |  |
| L1-16 | SRCM103622 | 7 | 6 | 20 | 11 | 14 | 25 | 14 | 4 |  |
| L1-17 | SRCM103773 | 11 | 13 | 19 | 10 | 13 | 17 | 16 | 4 |  |
| L1-18 | SRCM102753 | 4 | 14 | 8 | 11 | 21 | 18 | 18 | 4 |  |
| L1-19 | SRCM104008, SRCM104011 | 7 | 19 | 12 | 11 | 14 | 25 | 18 | 4 |  |
| L1-20 | SRCM103576 | 5 | 10 | 20 | 13 | 13 | 12 | 19 | 4 |  |
| L1-21 | SRCM103697 | 5 | 8 | 12 | 13 | 22 | 12 | 19 | 4 |  |
| L1-22 | SRCM101441, SRCM103886 | 5 | 9 | 20 | 13 | 22 | 12 | 19 | 4 |  |
| L1-23 | SRCM100757, SRCM100761, SRCM101444, SRCM103637, SRCM103641 | 11 | 9 | 19 | 11 | 13 | 21 | 19 | 4 |  |
| L1-24 | SRCM102745 | 7 | 16 | 18 | 11 | 9 | 21 | 20 | 4 |  |
| L1-25 | SRCM102754 | 10 | 15 | 16 | 13 | 10 | 13 | 17 | 5 |  |
| L1-26 | SRCM100333 | 5 | 10 | 19 | 14 | 20 | 11 | 19 | 6 |  |
| L1-27 | SRCM103612 | 9 | 16 | 9 | 9 | 22 | 17 | 19 | 6 |  |
| L1-28 | SRCM103581 | 12 | 17 | 20 | 11 | 12 | 10 | 19 | 7 |  |
| L1-29 | SRCM103551 | 7 | 10 | 14 | 10 | 9 | 17 | 8 | 8 |  |
| L1-30 | SRCM103571 | 4 | 20 | 7 | 8 | 16 | 22 | 17 | 8 |  |
| L1-31 | SRCM102756 | 6 | 7 | 19 | 11 | 11 | 19 | 15 | 9 |  |

The allele sequence of the gene identified in the P1 scheme can be identified in pubMLST (https://pubmlst.org/organisms/bacillus-subtilis), and the allele sequence of S1, S2, and L1 can be identified in Figshare .
