# Supplementary material for: Comparison of four multilocus sequence typing schemes and amino acid biosynthesis based on genomic analysis of Bacillus subtilis
Source: PLoS One. 2023 Feb 21;18(2):e0282092. doi: 10.1371/journal.pone.0282092 (PMC9943010; doi:10.1371/journal.pone.0282092)
Supplement: S3 Table — (DOCX) [file pone.0282092.s004.docx]

**S3 Table. Compositional characteristics of genes used in the four MLST schemes for *B. subtilis.***

| Scheme | Gene | Length (bp) | No. of  alleles | No. of  polymorphic sites | No. of  variable sites | *dN/dS* | Discriminatory  power |
| --- | --- | --- | --- | --- | --- | --- | --- |
| P1 | *glpF* | 384 | 13 | 11 | 5 | 0.3956 | 0.867 |
| S1 | *ilvD* | 471 | 15 | 16 | 5 | 0.4440 | 0.923 |
|  | *pta* | 414 | 16 | 16 | 3 | 0.4295 | 0.923 |
|  | *purH* | 399 | 18 | 35 | 2 | 0.4751 | 0.936 |
|  | *pycA* | 399 | 12 | 13 | 4 | 0.4383 | 0.883 |
|  | *rpoD* | 384 | 5 | 4 | 2 | 0.395 | 0.463 |
|  | *tpiA* | 420 | 13 | 14 | 4 | 0.4676 | 0.779 |
| S2 | *gyrA* | 2466 | 22 | 97 | 11 | 0.4460 | 0.947 |
|  | *gyrB* | 1917 | 22 | 75 | 6 | 0.4693 | 0.955 |
|  | *purH* | 1539 | 22 | 117 | 5 | 0.4404 | 0.955 |
|  | *glpF* | 825 | 21 | 25 | 6 | 0.4069 | 0.956 |
|  | *pycA* | 3447 | 23 | 112 | 13 | 0.4378 | 0.964 |
|  | *ilvD* | 1677 | 19 | 51 | 7 | 0.4211 | 0.945 |
|  | *rpoD* | 1116 | 8 | 12 | 5 | 0.4275 | 0.771 |
|  | *tpiA* | 762 | 13 | 15 | 2 | 0.4725 | 0.779 |
|  | *pta* | 972 | 23 | 34 | 3 | 0.4451 | 0.956 |
| S3 | *gyrB* | 567 | 15 | 18 | 3 | 0.5019 | 0.808 |
|  | *adk* | 552 | 11 | 12 | 4 | 0.4577 | 0.867 |
|  | *pycA* | 713 | 16 | 24 | 3 | 0.4566 | 0.922 |
|  | *pyrE* | 449 | 16 | 33 | 2 | 0.4746 | 0.909 |
|  | *sucC* | 464 | 13 | 13 | 2 | 0.4948 | 0.919 |
|  | *mutL* | 702 | 15 | 24 | 4 | 0.4390 | 0.895 |
|  | *aroE* | 735 | 20 | 33 | 4 | 0.3775 | 0.950 |
| L1 | *adk* | 438 | 11 | 10 | 3 | 0.4506 | 0.867 |
|  | *ccpA* | 517 | 15 | 20 | 3 | 0.4882 | 0.853 |
|  | *glpF* | 505 | 15 | 16 | 4 | 0.4001 | 0.897 |
|  | *gmk* | 415 | 9 | 6 | 2 | 0.5232 | 0.746 |
|  | *ilvD* | 497 | 14 | 19 | 4 | 0.4564 | 0.900 |
|  | *pur* | 477 | 19 | 32 | 2 | 0.4755 | 0.940 |
|  | *spo0A* | 544 | 13 | 13 | 4 | 0.4415 | 0.872 |
|  | *tpi* | 413 | 6 | 6 | 2 | 0.5088 | 0.362 |
